# Using mHealth to improve tuberculosis case identification and treatment initiation in South Africa: Results from a pilot study

**DOI:** 10.1371/journal.pone.0199687

**Published:** 2018-07-03

**Authors:** Noriah Maraba, Christopher J. Hoffmann, Violet N. Chihota, Larry W. Chang, Nazir Ismail, Sue Candy, Edwin Madibogo, Marc Katzwinkel, Gavin J. Churchyard, Kerrigan McCarthy

**Affiliations:** 1 The Aurum Institute, Johannesburg, South Africa; 2 School of Public Health, Faculty of Health Sciences, University of the Witwatersrand, Johannesburg, South Africa; 3 John Hopkins University School of Medicine, Baltimore, MD, United States of America; 4 National Institute of Communicable Disease, Johannesburg, South Africa; 5 Department of Health and Social Development, City of Johannesburg, Johannesburg, South Africa; 6 Mobenzi, Cape Town, South Africa; 7 Advancing Treatment and Care for TB and HIV, South African Medical Research Council Collaborating Center for HIV/TB, Johannesburg, South Africa; 8 London School of Tropical and Hygiene Medicine, London, United Kingdom; Médecins Sans Frontières (MSF), SOUTH AFRICA

## Abstract

**Background:**

Tuberculosis (TB) incidence in South Africa is among the highest globally. Initial loss to follow-up (ILFU), defined as not starting on TB treatment within 28 days of testing positive, is undermining control efforts. We assessed the feasibility, acceptability, and potential of a mHealth application to reduce ILFU.

**Methods:**

An mHealth application was developed to capture patients TB investigation data, provide results and monitor treatment initiation. This was implemented in two primary health clinics (PHC) in inner-city Johannesburg. Feasibility was assessed by comparing documentation of personal details, specimen results for same individuals during implementation period (paper register and Mhealth application). Effectiveness was assessed by comparing proportion of patients with results within 48 hours, and proportion started on treatment within 28 days of testing TB positive during pre- implementation (paper register) and implementation (mHealth application) periods. In-depth interviews with patients and providers were conducted to assess acceptability of application.

**Results:**

Pre-implementation, 457 patients were recorded in paper registers [195 (42.7%) male, median age 34 years (interquartile range IQR (28–40), 45 (10.5%) sputum Xpert positive]. During implementation, 319 patients were recorded in paper register and the mHealth application [131 (41.1%) male, median age 32 years (IQR 27–38), 33 (10.3%) sputum Xpert positive]. The proportion with complete personal details: [mHealth 95.0% versus paper register 94.0%, (p = 0.54)] and proportion with documented results: [mHealth 97.4% versus paper register 97.8%, (p = 0.79)] were not different in the two methods. The proportion of results available within 48 hours: [mHealth 96.8% versus paper register 68.6%), (p <0.001)], and the proportion on treatment within 28 days [mHealth 28/33 (84.8%) versus paper register 30/44 (68.2%), (p = 0.08)] increased during implementation but was not statistically significant. In-depth interviews showed that providers easily integrated the mHealth application into routine TB investigation and patients positively received the delivery of results via text message. Time from sputum collection to TB treatment initiation decreased from 4 days (pre-implementation) to 3 days but was not statistically significant.

**Conclusions:**

We demonstrated that implementation of the mHealth application was feasible, acceptable to health care providers and patients, and has potential to reduce the time to TB treatment initiation and ILFU in PHC settings.

## Introduction

There are an estimated 834 cases of tuberculosis (TB) per 100 000 population diagnosed annually in South Africa [1]. Without appropriate initiation of treatment, people with pulmonary TB may transmit TB to others and suffer from TB associated morbidity and mortality [2]. Initial loss to follow-up (ILFU) is defined as not starting TB treatment within 28 days of a microbiologically confirmed TB test. ILFU is estimated to occur among 17–25% of all persons diagnosed with TB in South Africa [3–5].

Common reasons for ILFU in TB patients include health system-, patient- and disease-related factors [6]. Health system-related reasons for ILFU include: delays in clinics receiving results or failure to receive results from laboratories; the need for patients to return repeatedly to clinics to enquire about results, and long waiting periods to receive care in health facilities. Patient-related factors include the need to take time off work to return to the clinic, and being prevented through illness from returning to clinic [6]. Optimal strategies to reduce ILFU will likely need to address both clinic-and patient-level factors.

Mobile technology use in health care, often referred to as mHealth, has potential to address both clinic and patient-level factors related to ILFU. mHealth has been used for communication with both providers and patients and has been reported to be acceptable and effective in diverse settings including during pre-natal screening, sexually transmitted disease care and HIV management [7–9]. mHealth innovations may help close patient- and health system-related gaps that contribute to ILFU. We therefore aimed to assess, using mixed methods, the feasibility, acceptability, and potential for addressing TB ILFU of a mHealth application to support and improve the TB care continuum.

## Methods

### Study setting

This study was based at two primary health clinics (PHC) serving high-TB-burden communities located in Johannesburg, South Africa.

### mHealth application development

**Fig 1:**
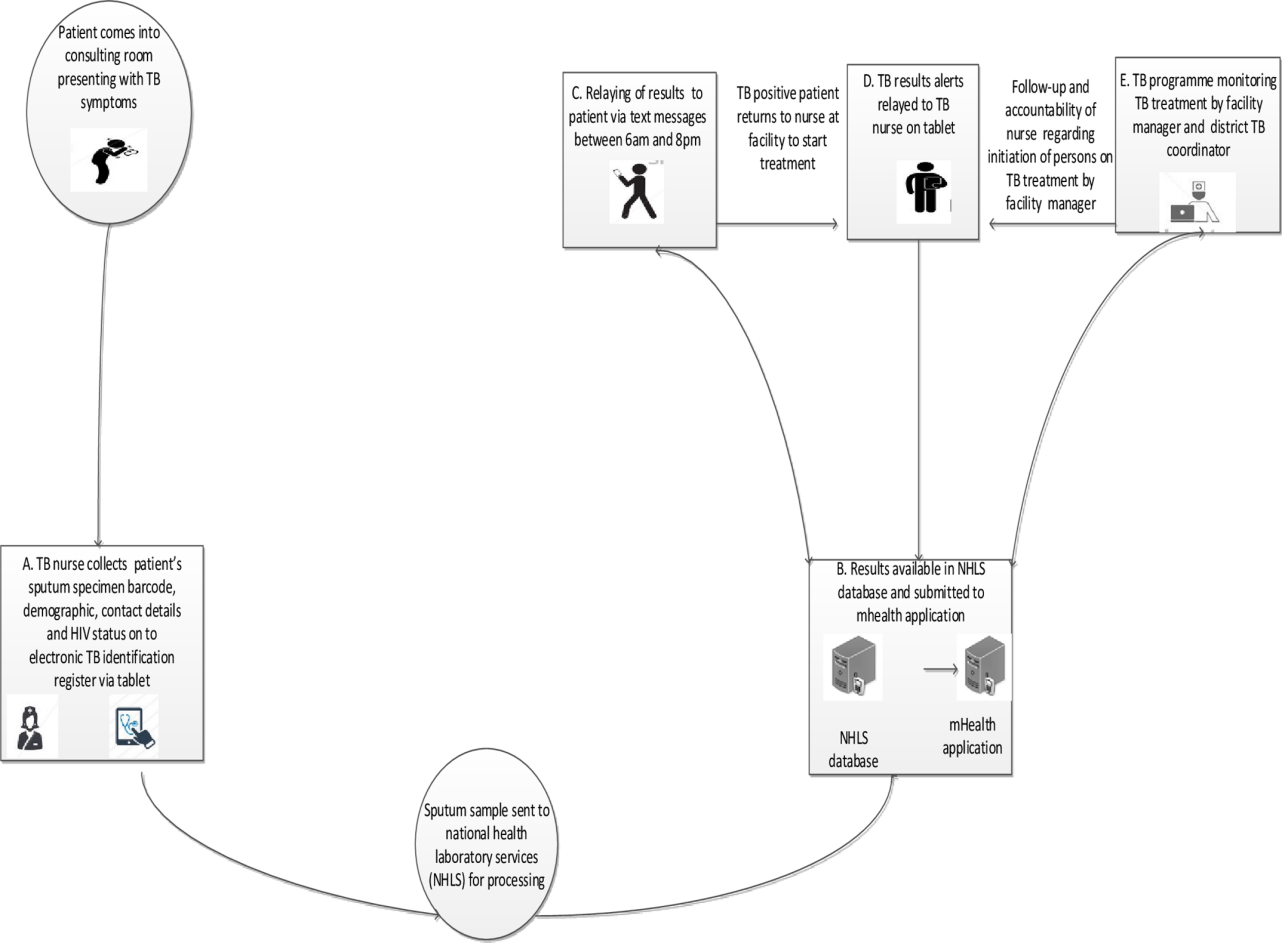
Illustration of the direction of communication between laboratory, health care worker and patient during TB case investigation. NHLS: National Health Laboratory Services; TB: Tuberculosis.

The mHealth application was developed by the study team in collaboration with the District TB Control Program (TCP), PHC managers and the National Health Laboratory services (NHLS). The mHealth application was designed to (1) replace the data collection and reporting function of a TB register through direct data entry onto a mobile application at the point of care of TB case identification data, (2) automate TB laboratory result delivery to PHC clinic by directly pulling results from a central lab (NHLS) into the mHealth system, (3) provide a dashboard display of the status of all patients in the TB testing and care continuum, (4) provide electronic notification of TB sputum results to clinic TB staff, and (5) provide pin-protected electronic TB sputum result notification to patients. The goal was to produce an application that would reduce the time and effort required for TB data reporting, provide rapid and automatic access to Xpert MTB/Rif TB test results and empower patients by directly providing results via cell phone messaging. The TB result-reporting component allowed health care workers to track patient progress to treatment initiation, prompt TB nurses to submit baseline smear microscopy on patients with positive Xpert MTB/Rif results and identify as well as remediate patients with a break in care (e.g. initiation of TB treatment for those with TB positive sputa) (Fig 1).

The content of results messages sent to patients was developed and refined during a workshop with the district TB coordinator, clinic TB nurses, and facility DOT supporters. Patients were notified by text of result availability, and were then required to access the results using a four-digit secret pin which patients chose at initial clinic visit. Patients who requested to receive an electronic result received the message ‘Please reply to this message with your secret pin in order to receive your MTB results’. If the participant entered the correct pin, one of the following messages were provided: 1) ‘Your result is MTB negative, please visit clinic <name> if symptoms persist’; 2) ‘Your result is MTB positive, please visit clinic <name> in order to start TB treatment’; or 3) ‘Your result is unsuccessful specimen, please visit clinic <name> to provide further specimens’. Patients who did not want to receive the results via text message but wanted to be notified of results availability received the message ‘Your MTB results are ready for collection at the clinic’.

### Study participants and procedures

The study was divided into two periods. The pre-implementation period occurred from January to March 2015. The clinic staff received training updates on use of the standard mHealth application and continued to use the paper register for all TB-related data collection. Prior to the implementation period, there was a ‘run-in’ period for training of study staff and to allow for troubleshooting of operational challenges encountered with the mHealth application. The implementation period occurred between February and April 2016. During the implementation period, study staff recruited sequential adults ≥18 years of age who had been referred for TB investigation including sputum testing. Patients were allowed to select whether to receive sputum results directly on their phone via text message or to get a text message informing them that their results were available at the clinic. Reasons for participant refusal to receive text messages were not collected. During the implementation period, clinic staff used the mHealth application for recording all patient data while in parallel; study staff captured the same data onto the paper registers. Simultaneous data collection on mHealth and paper registered allowed for comparison of data collection methodology whilst ensuring that the clinic adhered to TB control programme recording and reporting practice regarding the use of paper registers.

### Feasibility analysis

#### Data collection

Feasibility was assessed by evaluating the concordance of data captured in both the mHealth application by clinic staff and the paper-based register by research staff for the same set of patients in the implementation period. We decided *a priori* that the mHealth application would be feasible if we observed no difference between key indicators generated from the paper and mHealth applications as follows: in (i) successful collection by the mHealth application of all data elements usually collected by the paper TB register, (ii) successful and more rapid receipt of electronic laboratory results at the clinic using the mHealth application (iii) success in automatic sending of text notifications and results to patients. We compared the following indicators generated from data in the mHealth application and on the paper register during implementation: 1) the proportion of patients with specimen results; 2) proportion with positive Xpert MTB/Rif results; 3) the proportion of patients with a positive Xpert MTB/Rif result who also had a subsequent smear result recorded.

#### Data analysis

A McNemar test was used to compare indicators generated from mHealth and paper registers in implementation period. A statistically significant difference in indicators (as evidenced by a p-value <0.05) suggested a difference in data collection accuracy, thus allowing inferences to be made regarding feasibility of the mHealth application to support TCP monitoring and evaluation requirements.

### Acceptability

#### Data collection

Acceptability of the mHealth application to patients and healthcare providers was assessed using semi-structured interviews. At seven months post- implementation of the mHealth application, structured interviews were conducted with purposively selected patient participants who (1) tested TB negative and received the result via a text message, (2) tested TB negative and did not receive the result via text message, (3) tested TB positive and received the result via text message, or (4) tested TB positive and did not receive results via text message. Interviews were conducted in English, isiZulu, Sesotho or Setswana and lasted approximately 30 minutes. Structured interviews were conducted with providers who used the mHealth application at each clinic and the regional TB coordinator. All provider interviews were conducted in English. All interviews were recorded and transcribed and, when required, translated into English for analysis. Data were uploaded and analysed in Nvivo version 10 (QSR international Pty software).

#### Data analysis

We adapted Normalization Process Theory (NPT) [10], to frame our analysis of patient and provider interviews [10] [11]. We specifically focused on NPT domains of: (1) ‘cognitive participation’ which we defined as the provider’s willingness to capture patient’s data into the application and search for results, or the patient’s willingness to use the application to retrieve and receive results, (2) ‘collective action’ which we defined as: a) the usability of the mHealth application and how it integrates within existing systems; b) the ease of use of the mHealth application by providers and patients, and (3) ‘reflexive monitoring’ which we defined as the patient’s or providers’ experience in using of the device to retrieve results [12,13]. To the existing NPT domains, we added ‘confidentiality’ which we defined as patients’ confidence in the system’s ability to disclose their results appropriately. Using these domains, transcripts were coded by NM. Specific passages were discussed among the investigative team to resolve discrepancies in coding and identify key themes.

### Potential effectiveness

#### Data collection

Potential effectiveness was assessed through comparing specific indicators collected during the pre-implementation and implementation period data. We calculated TB treatment initiation within 48 hours. We chose TB treatment initiation within 48 hours as it is the cut-off point that the national TB programmes uses to describe ILFU [14] while a cut-off point of 28 days was chosen to allow comparison with prior studies from South Africa regarding ILFU [3–5,15].

#### Data analysis

Change in specific indicators related to timing of return of sputum results to clinic, proportion started on TB treatment within two and 28 days was assessed and proportion lost to follow-up. The proportion test for two samples was used to compare indicators generated from mHealth data in implementation period and from paper registers in the pre-implementation period. A statistically significant difference in indicators (p value <0.05) suggested that a real difference in proportions between the two methods exists. A Mann-Whitney test was done to compare for time to TB treatment in the pre-implementation and implementation periods.

## Ethical considerations

Approval to implement the study was provided by the institutional review boards for human subjects research of the University of the Witwatersrand (HREC certificate no: M141008) and Johns Hopkins University (IRB certificate no: IRB00071530). All participants provided written informed consent to take part in the study.

## Results

### Demographic characteristics

In pre-implementation period, 457 patients were recorded in the TB register. Of these patients, 195 (42.7%) were male, median age was 34 years (interquartile range [28–40]), and 353 (77.2%) patients self-reported being HIV positive (Table 1). In the implementation period, 319 persons were enrolled and registered on the mHealth application and paper register of whom 131 (41.1%) were male, median age 32 years (IQR 27–38), 259 (81.2%) self-reported being HIV positive. Of 319, 286 (89.6%) requested to receive results by text message, 31 (9.7%) did not wish to receive any text message and two (0.6%) opted to receive a text message indicating availability of results at the clinic. Of the 286 TB patients who requested to receive results via text messages, 59 (20.6%) failed to receive their results. This was due to:1) user failure for 49 (83.1%) patients who did not understand how to respond with a secret pin code; 2) data interface failure (the application did not receive results from the laboratory) for five (8.4%) patients; and 3) application failure (mHealth did not send results after patients responded with a correct pin) for five (8.4%) patients.

**Table 1:** Baseline characteristics of patients in pre-implementation and implementation period.

	Pre-implementation period	Implementation period
Variables	N (%)	N (%)
	N = 457	N = 319
**Gender**		
**Male**	195 (42.7%)	131 (41.1%)
**Age (median, IQR)**	34 (28–40)	32 (27–38)
**HIV status (self-reported)**	353 (77.2%)	259 (81.2%)
**Opted to receive results via text**		
**Yes**	N/A	286 (89.6%)
**No**	N/A	31 (9.7%)
**Text for availability of results**	N/A	2 (0.6%)

IQR: Interquartile range; N/A: not applicable

### Feasibility

During the implementation period we sought to determine if there was any loss in data quality through electronic data collection compared to paper registers. We found no statistically significant difference between indicators derived from the mHealth application versus those obtained from paper-based registers as follows: 1) the proportion with complete personal data (mHealth 95% versus 94% paper register); 2) the proportion with a recorded specimen result (mHealth 97.4% versus 97.8% paper register); 3) the proportion with Xpert MTB/RIF positive result (mHealth 10.3% versus 10.6% paper register). (Table 2). The proportion of Xpert MTB/RIF positive TB results with a subsequent sputum smear result (mHealth 84.8% versus paper register 52.9%) was significantly higher with the mHealth application (Table 2).

**Table 2:** Feasibility of documenting data using the mHealth application compared to paper register.

	Implementation period		
	Paper register	mHealth	P value
Process indicators	N (%)	N (%)	
**Total population screened by health care workers at clinics using TB screening tool**	N = 319	N = 319	
**Number with complete personal details**	300/319 (94.0)	303/319 (95.0)	0.54
**Number with a recorded specimen result**	312/319 (97.8)	311/319 (97.4)	0.79
**Number with Xpert® MTB/RIF positive result**	34/319 (10.6)	33/319(10.3)	0.89
**Number of positive, Rif sensitive Xpert® MTB/RIF results with a baseline specimen submitted for** **smear microscopy**	18/34 (52.9)	28/33 (84.8)	<0.001

TB: Tuberculosis; Rif: Rifampicin

### Acceptability

Qualitative interviews were conducted with 21 TB patients (11 with positive sputum for TB and 10 with negative TB sputum results) and eight healthcare workers.

Cognitive participation: The majority of patients (n = 13) valued the convenience of the mHealth application allowing them to access results without having to visit the clinic (Table 3, Quote 1). The majority of providers believed that the mHealth application assisted patients to start TB treatment earlier (Table 3, Quote 2 and 3).

**Table 3:** Patients and provider experiences of the mHealth intervention for TB case investigation.

Evaluation domains	Quote
**Cognitive participation**	***QUOTE 1***:*“Yes I would recommend it*, *at least it saves you time than you coming to the clinic standing in a queue and you will get the same result as the one you will get on your phone*. *When you get it on your phone*, *you at home*, *you do not leave*, *or when you are at work*, *and sometime at work you cannot get day offs just to come and collect results*. *And when you come to the clinic to collect your result you must come in the morning*. *You see*, *it clashes with the time to work*. *When you get via a text message it is easy*, *you can go to work and still get your result*.*”* ***Female* TB negative patient no 5, clinic B**
	***QUOTE 2*:** *“I strongly recommend it because we have a community that moves around a lot*, *so it will help because people leave the sputum then go to KZN* [Kwazulu Natal Province]. *If they have this system in place*, *they would go to the nearest clinic and show them the result and start them on treatment”*. **Female, facility manager clinic B** ***QUOTE 3***: *“Yes* [I would recommend it]. *It will make TB rooms work much easier*. *If it can be something that is used permanently in all the clinics it can make everything easier*. *It really makes things easier; we do not have to wait for hard copy because the printing of results delays sometimes and you can get the results after 2 days*. *But this one* [text message intervention] *you get your results within 24hrs like I send a sputum today and tomorrow morning the results are back I initiate the patient without me waiting for hardcopy which can take even 3 days because a human error happens there courier takes the results to another or wrong clinic then it delays the process of starting TB treatment”*. **TB nurse, Clinic A**
**Collective action**	***QUOTE 4*:** *“we did experience challenges in the beginning because the tablet scanner was not able to capture barcode and it was delaying us*. *Then we reported it to the research team leader who acted fast and they got us a manual scanner and …*..*capturing of barcode was faster“*. **TB nurse, clinic B**
	***QUOTE 5*:** “*yes I got the text message*, *…*.*the sister said when I get a text message*, *she gave me these other numbers* [the pin code], [and] *she said I must send to that number*. *She showed me how*. *I did what she said*, [but] *I did not get* [result], *so I never got the result*.*”* **Male TB negative patient 3, clinic B**
	**QUOTE 6**: *“They sent them* [results] *but I was not responding in a good way until I came to the clinic and they assisted me in getting the results*, *I pressed wrongly on my phone*.*”* **Male TB positive patient no 3, clinic B**
	**QUOTE 7**: *“text message I got it*, *but*, *I am not well educated*, *I asked the ones that are*, *they said I did not have TB*, *but I may have it*, *they will follow on it*.*”* **Male TB negative patient no 3, clinic B**
	**QUOTE 8:** *“Most of the patients were coming back before I call them or send a tracer*, *if their results were positive*.*”* **TB nurse, Clinic B**
	**QUOTE 9:** *“The person I saw here when I arrived treated me well a nurse that work here in TB room*, *I told her I have received the results and the text saying I have got TB then she said I must wait next to Tb room since there was someone in the room then I will get in when they leave*, *I did not even take long maybe ±20 to 30 minutes to finish*, *I got helped very quickly*.*”* **Male TB positive patient 5, Clinic A**
**Reflexive monitoring**	***QUOTE 10***: *“It* [the text message intervention] *help that client came quicker to start treatment*, *they didn’t delay in coming*, *others even before we could think they would be here they were already in the clinic to start treatment and those who were lost to follow-up we could immediately see that these need to be contacted so that they can come and start treatment*.*”* **Facility manager, Clinic A**
	***QUOTE 11***: *“Patients were satisfied because some of them did not have to ask for permission* [from work] *to come to the clinic more especially the ones that the results come back negative*.*”* **Facility manager, Clinic B**
**Confidentiality**	***QUOTE 12***: *“sister like I know my phone it’s mine*, *it’s my private inside*, *even if I get a text I will open it and see it myself*, *and no one will know what is happening on my phone*, *I think it’s a good way to receive your result through text through the phone*. *Than to come to the clinic sister*, *and for the sister to see it*. **Male TB negative patient no 2, clinic B**

Collective action: Some providers (n = 4) expressed concerns about technical difficulties experienced while using the mobile devices during the initial phase of implementation. However, they indicated that the use of technology improved with experience (Table 3, Quote 4). Once providers gained experience using the application they reported that it assisted them to start patients on TB treatment because patients returned to the clinic promptly after receiving results on their cell phones (Table 3, Quote 8). Some patients (n = 6) also reported that they encountered technical challenges that prevented them from retrieving their results (Table 3, Quote 5). At times, this arose through software malfunction that failed to deliver results despite patients correctly following retrieval instructions. Individual comprehension of steps also interfered with retrieving results (Table 3, Quotes 6 and 7). Patients who received results reported that it facilitated their return clinic visit (Table 3, Quote 9).

Reflexive monitoring: Overall, the majority of interviewed providers (n = 7) and patients (n = 14) were satisfied with the intervention. Providers perceived that the mHealth application assisted with patients returning to the clinic faster to start on TB treatment and saved patients’ time (Table 3, Quote 10 and 11).

Confidentiality: Some patients (n = 7) raised the matter of confidentiality of receiving results through text messaging and six of them appreciated receiving results via pin-protected text message (Table 3, Quote 12). There were no reports of inadvertent disclosure of results.

### Potential effectiveness

The largest improvement we observed in implementing the mHealth application was the proportion of TB results documented at the clinic within 48 hours when comparing the observation period immediately prior to mHealth use and the mHealth implementation period. During the pre-implementation period 68.6% of TB results were documented at the clinic within 48 hours compared to 96.8% in the implementation period (Table 4). Time to TB treatment was decreased from 4 days (IQR 2–6) in the pre-implementation period to 3 days (IQR 2–5) in the implementation period; this was not statistically significant (p = 0.5).

**Table 4:** Process indicators assessing potential effectiveness of using mHealth application compared with paper register.

	Pre-implementation	Implementation	
Source of data	Paper register	mHealth	P value
Process indicators	N (%)	N (%)	
**Number with turn-around time for sputum results within 48 hours**	293/427 (68.6)	309/319 (96.8)	<0.001
**Time to TB treatment, median (IQR)**	4 (2–6)	3 (2–5)	0.50
**Number on treatment within 48 hours of testing Rif susceptible TB positive**	14/44 (31.8)	10/33 (30.3)	0.85
**Number on treatment within 28 days of testing Rif susceptible TB positive**	30/44 (68.2)	28/33 (84.8)	0.08
**Number lost to follow-up (not on treatment within 56 days)**	14/44 (31.8)	5/33 (15.2)	0.08

TB: Tuberculosis, Rif: Rifampicin, IQR: Interquartile range

## Discussion

This pilot study demonstrated that our mHealth intervention to improve the TB care was feasible, acceptable and appeared to offer advantages over the paper based register. The study showed that the mHealth TB evaluation and treatment management application effectively supported all paper register functions while appearing to improve data quality and to decrease time to TB treatment initiation.

One of the reasons why the mHealth application reduced the time for patients to start TB treatment is that patients returned to the clinic when they were notified of result availability. This eliminated the time required by the laboratory to hand-deliver printed sputum results to the clinic, and the time required for TB nurses to sort the paper results and call patients with positive results. The fact that patients did not need to return to clinic until notified that results were available also has potential to considerably lessen the financial and time burden on those who test TB negative. It has potential to reduce the workload on TB nurses as fewer patients with negative results would need to return to the clinic. Further, we observed that patient’s knowledge of their sputum results enhanced their self-efficacy: for example, patients who tested positive for TB arrived at the clinic requesting TB treatment. An additional reason for improved time to receipt of results and TB treatment initiation is that our mHealth application eliminated the administrative work required to locate patients’ register entries and file their paper-based results as healthcare workers could rapidly locate patient’s results on the tablet. Our findings are similar to a study done in Swaziland that delivered sputum TB results via text message to clinics and showed an improved turnaround time to TB treatment initiation compared to receiving results via paper reports [16].

Improvements in indicators when comparing the mHealth application to the paper-based register likely represents valid and meaningful changes. For example, the mHealth application obtained TB lab results through a direct data download from the national laboratory system server through a matching process based on the specimen identification barcode. Therefore the mHealth register contained the “true” result reported in the national database and eliminated potential for human transcription error. In contrast, sputum results were written into the paper register by a health care worker after: (1) a hard copy of the laboratory results were received at the clinic, AND (2) the health care worker appropriately filed the results, AND (3) the health care worker correctly transcribed the data into the register. Each of these three steps was open to system or human error. Regarding the proportion of TB positive patients on treatment by 28 days after testing, this reflects the date of treatment commencement as recorded by health care worker in either the register or the mHealth application. Data entry into the register or the mHealth application was subject to same risk of error, however, the mHealth application date entry field included inbuilt validity checks to reduce incorrect date entry (E.g. selecting today’s date as the date of visit; and preventing visit dates out of a set range).

Implementation of the application was not without challenges. Patient-level challenges were related to lack of proficiency in receiving text messages, in understanding the content of the messages and in replying to text messages with their secret pin. Patient-level challenges with retrieving laboratory results through mobile applications have been reported in other studies and were ascribed to literacy levels and familiarity with using a mobile phone [17]. In a study done in Uganda, persons who were not concerned about confidentiality of receiving results via text message and who were illiterate approached a person they trusted to read their results for them [18]. However, these patient-level challenges may be overcome with improved patient instruction or instructional aids. In addition, proficiency in the use of mobile phone applications will improve as society increasingly adopts mobile technology. This, along with the qualitative satisfaction in the use of the application by the majority of our participants, suggests that the added value of the application outweighs obstacles created by patient-level challenges.

This was a pilot study in two clinics using the mHealth application in parallel with the TCP paper case investigation register. Thus, the generalizability to wider use and to system deployment in the absence of a paper-based register cannot be directly inferred from the study. However, we believe that dispensing with the paper register will increase efficiency without compromising care or programmatic TB reporting. Notably, the mHealth application was able to sustain TB evaluation and management in two busy public sector primary care clinics whilst simultaneously being fully operational by public sector staff.

In conclusion, we have demonstrated that the mHealth application tested in this study may take the place of current paper-based TCP case-finding tools, and may have potential to substantially reduce the time to TB treatment initiation, improve TCP management, identify persons with delay in TB treatment initiation and empower patients with direct receipt of TB results. Implementation of an mHealth application to support and improve the TB care has the potential to substantially improve treatment indicators and patient-level outcomes and large-scale evaluations of this technology are urgently required.

## Supporting information

S1 Table Minimal dataset for variables reported in the pre-implementation period. (CSV)

S2 Table Minimal dataset for variables reported in the implementation period. (CSV)

S1 Text Data dictionary–Variable names, description and value labels for pre-implementation period. (TXT)

S2 Text Data dictionary–Variable names, description and value labels for implementation period. (TXT)
